# Predictive value of aspartate aminotransferase and alanine aminotransferase levels in vaginal fluid for the diagnosis of premature rupture of membranes

**Published:** 2014-04

**Authors:** Maryam Asgharnia, Fariba Mirblouk, Fatemeh Salamat, Babak Ashrafkhani, Zahra Dirbaz

**Affiliations:** 1 *Reproductive Health Research Center, Department of Obstetrics and Gynecology, Guilan University of Medical Sciences, Rasht, Iran. *; 2 *Guilan University of Medical Sciences, Rasht, Iran.*

**Keywords:** *Aspartate aminotransferase*, *Alanine aminotransferase*, *Premature rupture of membranes*

## Abstract

**Background:** Preterm premature rupture of membranes (PPROM) occurs in 3% of pregnancies and 30-40% of preterm labors are related to this problem. Early diagnosis of PPROM is very important due to its impact on pregnancy outcomes. **Objective:** To determine the diagnostic value of aspartate aminotransferase (AST) and alanine aminotransferase (ALT) levels in vaginal fluid for the diagnosis of preterm premature rupture of membranes as a non-invasive and available test.

**Materials and Methods: **A total of 148 pregnant women between the 26^th^-36^th^ gestational weeks were enrolled in the study. 74 patients were in PROM group and 74 in control group. AST and ALT levels in vaginal fluid were measured in each group. Mann Whitney U-test was used to compare AST and ALT levels in each group.

**Results: **The mean of AST level in vaginal fluid was 12.77±10.06 in PROM group vs. 6.91±10.92 in control group (p<0.001), while there were no significant difference between ALT levels in PROM group 1.51±3.17 and control group 0.89±1.15 (p=0.49). Optimal cut point of AST for the diagnosis of PROM was 4.5 IU/L in this study. The sensitivity, specificity, positive and negative predictive values were 82.4%, 63.5%, 69.32% and 78.33% respectively.

**Conclusion:** According to the findings of this study, measurement of AST level in vaginal fluid can be used as a reliable test for diagnosis of PROM, but there is no good cut point for ALT level that can be practically used.

## Introduction

Preterm birth is defined as the birth of an infant prior to 37 weeks of gestation which accounts for a large proportion of causes of infant mortality and morbidity. Premature water breaking, also known as preterm premature rupture of membranes (PPROM), is one of the most prevalent midwifery problems associated with 30% or 40% of premature births (1). Some of the possible causes of PPROM are: infection, cigarette smoking, too much amniotic fluid (hydramnious), multiple futeses, nutritional deficiencies, previous PPROM, cervical incompetence (cervical length of less than 2.5 cm), and vaginal bleeding during early pregnancy (2). PPROM increases the mortality rate in both mothers and infants and the dangerous complications that may occur with it include umbilical cord prolapse, chorioamnionitis, sepsis and early delivery (2-4). 

Since there are many fetal complications resulting from long-term rupture of membranes and preterm delivery, early diagnosis of the rupture is very important and the frequent positive and negative cases, according to Fern and Nitrazine tests, have led to the discharge of biochemical markers to diagnose the rupture. PPROM diagnosis is through physical findings such as direct visualization of amniotic fluid in posterior fornix, Fern test and sonography (done to assess AFI) and Nitrazine; none of which are completely valid (6, 7). 

The diagnosis is very difficult 48 hours or more after PPROM, and, in most cases, positive or negative results of Fern or Nitrazine tests are meaningless. Since misdiagnosis or lack of immediate diagnosis can cause serious pregnancy complications, and false positive results can, also, lead to inappropriate intervention, it is necessary to perform any biological tests to determine the results accurately and quickly. So far, several markers such as Prolactin, β human chorionic gonadotropin (βHCG), Calcitropic hormone, lactate dehydrogenase hormone (LDH), Creatinine (CR), etc. in vaginal fluid have been examined in diagnosis of ruptures of the membranes; however, they have had limited success in PROM diagnosis (4, 5, 8-12).

Liver enzymes such as AST (Aspartat Amino Transferase) and ALT (Alanine Amino Transferase) are generated by fetus and secreted in amniotic fluid and there is no relation between their amount and that of maternal enzymes. The intensity of AST and ALT in amniotic fluid increases by the increased gestational age. The intensity of these enzymes in amniotic fluid has been shown in various studies, but, until now, there is not any fixed and accurate information about the level of these enzymes in washing vaginal fluid. The present research is a cross-sectional study with control group which is carried out to compare the results of AST and ALT enzymes measurement in vaginal washing fluid of PPROM group with those of normal one.

## Materials and methods

The study was based on pregnant women between 26-36 weeks of gestation with suspected pre-term labor who referred to Al-Zahra Teaching Hospital between March 2011 to Feb 2012 in Rasht, Guilan, Iran after approving by the Ethical Committee of Guilan University of Medical Sciences; the simple random sampling based on inclusion/exclusion criteria was done. Exclusion criteria included fetal distress, fetal anomaly, multiple pregnancy, preeclampsia, maternal liver disease, vaginal bleeding, vaginal infection, structural malformation of the fetus, placenta previa, and maternal pregnancy complications. 

Pregnant women at 26-36 weeks of gestation who did not have the mentioned problems or any vaginal infection entered the study. The case group consisted of the women with obvious rupture of membrane that confirmed by sterile speculum, for this group, sampling was done when no visible leakage was evident. Additional information including gravidity, parity and gestational age were collected by asking questions. The cost of lab tests was not taken into consideration. The personal information of the patients was kept secret, and all the patients signed an informed consent and recruited to the study consciously.

The diagnosis of membrane rupture was based on using speculum or observation of fluid secretion out of cervical canal. In order to measure AST and ALT levels of the case group (after leakage stoppage) and control group (during hospitalization), a 3cc sterile saline was used for washing their vagina and the fluid was pulled out by a syringe and sent to laboratory at Al-Zahra Hospital (Rasht); Pars Azmoon kits (as normal serum kits) were used for test measurements. This study was done with financial support of Vice Chancellor of Research, Guilan University of Medical Sciences.

**Statistical analysis**

In order to compare the mean levels of AST and ALT enzymes in two groups, Mann-Whitney U test was used, and ROC diagram was drawn using the AST and ALT levels for the diagnosis of preterm premature rupture of membrane, and sensitivity and specificity were calculated for different cut-off points. By considering the highest sensitivity and specificity levels, optimal cut-off points were reported for AST and ALT enzymes. Analysis of data was performed by using SPSS version 16 software.

## Results

A total of 148 pregnant women between 26 and 36 weeks’ gestation were recruited to the study, 74 women were taken as case group with observed rupture of membrane and 74 as control group without any observed rupture of membranes. The mean gestational age in this study was 33.39±2.33 weeks (range: 26-36), while the mean maternal age was 27.58±5.84 years (range: 15-45) (Table I). The mean gestational age was 33.33±2.56 weeks in case group, and 33.44±1.98 weeks in control group with no significant difference between them (p=0.78), and there was no significant difference between mean maternal age of case group (28.28±5.26) years and that of control group (26.89±6.32) years (p=0.148). Mann-Whitney U Test revealed that there was a statistically significant difference regarding AST measurement from vaginal washing fluid between cases and controls (p<0.001). 

**Figure 1:**
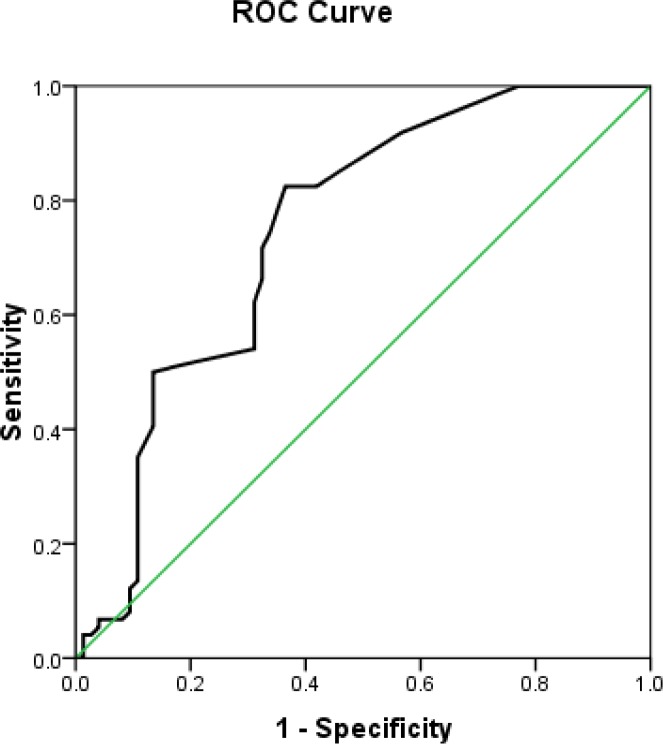
ROC curves for AST for the detection of PPROM. The area under ROC curve was 0.747.

**Figure 2:**
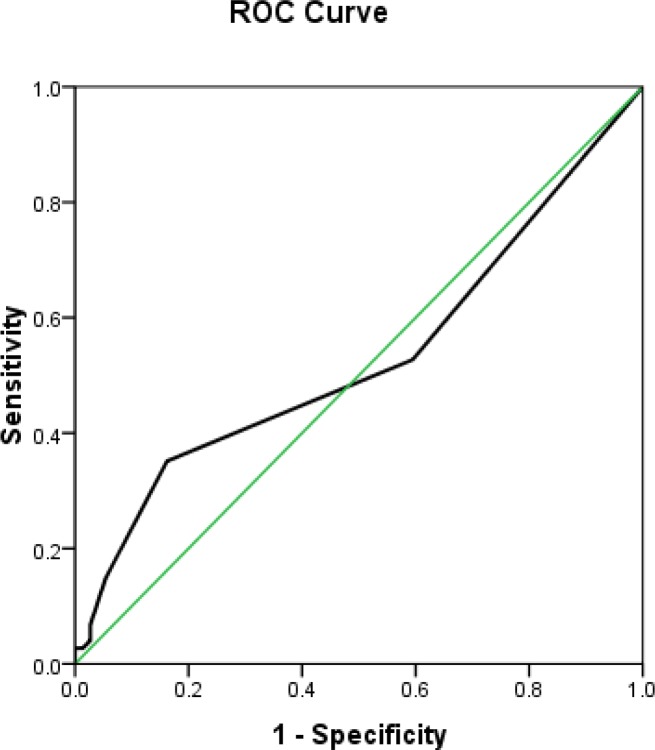
ROC curves for ALT for the detection of PPROM. The area under ROC curve was 0.53

There was no significant difference between ALT levels in two groups, 1.51±3.17 and 0.89±1.15 respectively (P=0.49) (Table II). The data from study revealed that the sensitivity and specificity, positive predictive value and negative value for the detection of AST were 82.4%, 63.5%, 69.32 and 78.33 respectively. After measuring the specificity and sensitivity of AST levels from vaginal washing fluid in the studied pregnant women, and evaluating the results presented in a ROC diagram for prediction of membrane rupture (Figure 1, 2) it was concluded that in this study the best cut point for AST was 4.5.

**Table I:** the frequency distribution of demographic and individual characteristics of pregnant women depending on the status of membrane

**Variables**	**Normal pregnancy(mean±SD)**	**PPROM group (mean±SD)**	**p-value**
Gestational age (weeks)	33.44 ± 1.98	33.33 ± 2.65	0.78
Maternal age (years)	26.89 ± 6.32	28.28 ± 5.26	0.148

* T-test

**Table II:** Comparison between AST and ALT levels in vaginal washing fluid samples of patients with PPROM and those of the control group

**Variable**	**Normal pregnancy**	**PPROM group**	**p-value**
ALT	0.89 ± 1.15	1.51 ± 3.17	0.49
AST	6.91 ± 10.92	12.77 ± 10.06	0.001

* Mann-Whithney U Test                    N=74.

Diagonal segments are produced by ties.

## Discussion

The current study was a cross-sectional study with a control group which was performed on 148 pregnant women between 26-36 completed weeks of gestation. The study did not show significant differences in gestational age and maternal age of the two groups as in other studies. In our study, there was a significant difference between the AST level of PPROM group and that of the normal group, but ALT levels showed no difference. Many studies for other biochemical markers in vaginal fluid such as βHCG, LDH, Urea, CR, lactate concentration and thyroid tests have been done and with the exception of Urea and CR, all of them maintained the same specificity and sensitivity values which were in agreement with the results of our study (1, 3, 5, 12, 13). However in comparison with other tests AST is economically cheaper. 

According to an article by Martinez *et al* the reported sensitivity and specificity for the Fern test ware 62% and 96%, respectively. In our study specificity of AST assessment was 82% which was 20% higher than the obtained results in Fern test. Therefore, measurement of AST level in vaginal fluid proved to be more useful for the diagnosis of PPROM and reduction of the rate of the undiagnosed PROM and its possible complications. In that study the diagnostic power of Fern test was 96% and higher than our study. However, Fern test requires a microscope and an experienced person to analyze the lamella which cannot easily be found everywhere (14). 

In a research done by Movahed *et al* on the diagnostic value of LDH and Creatinine in vaginal fluid, the gestational age was reported between 28 and 32 weeks which was different from the gestational age in our study (between 26-36), and this difference was the main reason for the different results(difference in sensitivity and specificity). The analysis of vaginal creatinine in that research showed a diagnostic value with sensitivity of 72% and specificity of 35% which was lower than the values in our study (AST sensitivity 82%, specificity 65%). Mean while, the sensitivity level of the LDH measurement in vaginal fluid revealed that the sensitivity levels in two test results were close and approximately equal (85% vs. 82%), whereas the specificity level of LDH test was higher than that of our study (80% vs. 65%), and this indicated that the number of the people without any membrane rupture confirmed by LDH test was more than the number confirmed by AST test in our study (higher specificity for LDH than AST) (1).

Ebro Cale *et al *reported that vaginal fluid ALT level was not statistically significant in PROM group as compared with control group but vaginal AST level were significant in the PROM group (6). The out off value of AST levels in the prediction of PROM was calculated as 3IU/L. the sensivity, specificity and positive and negative predictive values, of AST levels for PROM was 91, 83, 80 and 93% respectively. Despite that the gestational age was similar in two studies but their result was different, it may be due to smaller sample size in that study (6).

In one study carried out to measure βHCG level of vaginal fluid in diagnosis of premature rupture of membranes, the sensitivity level was 68% (lower than the result in our study 82%); however, the specificity of βHCG for the diagnosis of healthy cases was 95% and more than the result of AST test (3). Comparing the results of several studies, done over years in this field, with the results of present study revealed that whereas the sensitivity levels in different tests such as AST were almost similar and ranged from 80-90%, the specificity level showed different values, the highest of which belonged to βHCG ranging from 95-96% (3).

According to these results, and since there are too many human and financial costs of early detection, we should use the best types of tests and facilities to identify PPROM. AST is very economical and has high sensitivity and specificity; however, there are some other markers like βHCG which show great advantages of easy diagnosis. The weakness of current study was possible laboratory mistakes and the use of serum kits which might not have the right accuracy for vaginal washing fluid.

## Conclusion

Our data suggested that vaginal AST measurement in patients with PROM might be a useful marker as a predictive test. It´s place relation to other available tests needs examination in other larger studies. Also this issue has to be confirmed by further studies if an economical cost-benefit balance is to be taken into consideration.

## Footenote

***This article extracted from M.D. thesis. (Zahra Dirbaz)***
